# Equity assessment of maternal and child healthcare benefits utilization and distribution in public healthcare facilities in Bangladesh: a benefit incidence analysis

**DOI:** 10.1186/s12963-023-00312-y

**Published:** 2023-09-05

**Authors:** Nurnabi Sheikh, Marufa Sultana, Abdur Razzaque Sarker, Alec Morton

**Affiliations:** 1 Department of Management Science, Strathclyde Business School, University of Strathclyde, Glasgow, UK; 2 Health Economics and Health Technology Assessment (HEHTA), School of Health and Wellbeing, University of Glasgow, Glasgow, UK; 3 Deakin University, Institute for Health Transformation, Deakin Health Economics, School of Health and Social Development, Faculty of Health, Geelong, Australia; 4 Health Economic and Financing Research, Population Studies Division, Bangladesh Institute of Development Studies (BIDS), Dhaka, Bangladesh; 5 Saw Swee Hock School of Public Health, Tahir Foundation Building, National University of Singapore, 12 Science Drive 2, #10-01, Singapore, 117549 Singapore; 6 Health Economic Unit, Institute of Applied Health Research, University of Birmingham, Birmingham, UK; 7 Health Economics Unit, Melbourne School of Population and Global Health, University of Melbourne, Melbourne, Australia

**Keywords:** Benefit incidence analysis, Equity in health, Public spending, Maternal and child health, Universal health coverage, Bangladesh

## Abstract

**Background:**

The distribution of healthcare services should be based on the needs of the population, regardless of their ability to pay. Achieving universal health coverage implies first ensuring that people of all income levels have access to quality healthcare, and then allocating resources reasonably considering individual need. Hence, this study aims to understand how public benefits in Bangladesh are currently distributed among wealth quintiles considering different layers of healthcare facilities and to assess the distributional impact of public benefits.

**Methods:**

To conduct this study, data were extracted from the recent Bangladesh Demographic and Health Survey 2017–18. We performed benefit incidence analysis to determine the distribution of maternal and child healthcare utilization in relation to wealth quintiles. Disaggregated and national-level public benefit incidence analysis was conducted by the types of healthcare services, levels of healthcare facilities, and overall utilization. Concentration curves and concentration indices were estimated to measure the equity in benefits distribution.

**Results:**

An unequal utilization of public benefits observed among the wealth quintiles for maternal and child healthcare services across the different levels of healthcare facilities in Bangladesh. Overall, upper two quintiles (richest 19.8% and richer 21.7%) utilized more benefits from public facilities compared to the lower two quintiles (poorest 18.9% and poorer 20.1%). Benefits utilization from secondary level of health facilities was highly pro-rich, while benefit utilization found pro-poor at primary levels. The public benefits in Bangladesh were also not distributed according to the needs of the population; nevertheless, poorest 20% household cannot access 20% share of public benefits in most of the maternal and child healthcare services even if we ignore their needs.

**Conclusions:**

Benefit incidence analysis in public health spending demonstrates the efficacy with which the government allocates constrained health resources to satisfy the needs of the poor. Public health spending in Bangladesh on maternal and child healthcare services were not equally distributed among wealth quintiles. Overall health benefits were more utilized by the rich relative to the poor. Hence, policymakers should prioritize redistribution of resources by targeting the socioeconomically vulnerable segments of the population to increase their access to health services to meet their health needs.

## Introduction

The World Health Organization (WHO) stresses the importance of equitable health financing and healthcare delivery for a sustainable health system [1]. In the era of Sustainable Development Goals (SDGs), particularly for lower-middle income countries (LMICs), equitable access to healthcare is a major priority for health systems pursuing Universal Health Coverage. Being a LMIC, Bangladesh has made remarkable progress in many of its national and global health indicators mostly in maternal and child health over the past few decades [2, 3]. For instance, between 2001 and 2016, maternal mortality rate (MMR) declined significantly from 322 to 196 per 100,000 live births, and child mortality from 133 to 46 deaths per 1000 live births between 1989 and 2014 [2, 4]. Despite these progresses in national health indices, Bangladesh remains confronted with obstacles in ensuring an equitable distribution of health resources. Consequently, similar to in many other developing countries, there is a disparity in health outcomes across wealth quintiles.

The health system of Bangladesh is pluralistic in nature. The Government of Bangladesh (GOB) plays the key role along with the support of large private sectors, non-government organizations (NGO), and the donor agencies [1]. The Ministry of Health and Family Welfare (MOHFW) leads the public health systems that includes two divisions—(a) Health Services Division and (b) Medical Education and Family Welfare Division [5]. Bangladesh is one of the few countries that provides subsidized healthcare services through a well-structured health system with three tiers of healthcare facilities—primary, secondary, and tertiary. Primary-level facilities are the lower-level of health facilities located at the ward, union and upazila levels (lower geographical units) including community clinics (CC), union subcentre (USC), union health & family welfare centre (UH&FWC), and upazila health complex (UHC). Upazila health complexes provide both inpatient and outpatient services, whereas most of the other primary-level facilities are based on outpatient services. Secondary and tertiary-level healthcare facilities provide more advanced and specialized healthcare services [6]. Primary-level health facilities are inferior to secondary and tertiary-level facilities due to a lack of adequate resources including insufficient health care professionals, high absenteeism, and equipment shortage [6].

Out-of-pocket expenditure (OOPE) is the major source of health financing in Bangladesh which contributes to the woeful levels of inequity, and is also responsible for unequal healthcare service utilization in Bangladesh [7]. In addition, healthcare services offered by the private sector are costly and consequently inaccessible for most of the poor population, which contributes to disparity in access to healthcare services. The financial barrier for accessing healthcare is a persistent challenge for Bangladesh towards universal health coverage. A need-based allocation of direct health subsidies could be a way for reducing wealth disparities in Bangladesh if the poor are able to gain more health benefits from the subsidized public healthcare facilities. Equitable health access for all is vital because it is directly related to productivity, absenteeism, labour force participation, and hence economic growth [8]. Allocation of public health resources should be based on both efficiency and equity to optimise effectiveness of public spending and to meet the global universal health coverage agenda [9]. Therefore, national and international organizations emphasize the needs of studying the distributional impacts of public health spending.

Benefit Incidence Analysis (BIA) is an established method to assess the distributional impact of public spending, and to observe which wealth quintiles getting more benefits from public subsidies [10]. The method covers components of supply and demand for public healthcare services and analyses inefficiencies and disparities for public funding [9]. It also addresses the policy concerns about how effectively the health system is performing in targeting socioeconomically vulnerable people for public benefits. BIA has been used widely in many of the developing countries including India, Pakistan, Nigeria, Vietnam, Ghana, and Zambia to generate evidence on how healthcare services or benefits are allocated among wealth quintiles because of its ease of use and interpretation [9, 11–14]. A study conducted in Bangladesh has applied BIA to focus on public, private and NGO healthcare services and to address how benefits and the extent of benefits from different providers are distributed across wealth quintiles [15]. That study reported healthcare benefits in Bangladesh are pro-rich particularly for the benefits from private providers. In our study, we focused only on the public health facilities, particularly for the maternal and child healthcare services. However, we would like to explore more about how benefits are utilized across the various levels of public facilities. This facility-level BIA will enable policymakers to make decisions on which level of facilities they should invest more to make equitable health system in Bangladesh in terms of accessibility and affordability.

In case of Bangladesh, a common hypothesis is that poor people utilize more healthcare from public health facilities due to high OOPE in private sectors [16]. Hence, we intended to test this commonly used hypothesis on maternal and child healthcare services by utilizing nationally representative survey data. The objective of this study is to investigate public healthcare utilization pattern on maternal and child healthcare services among wealth quintiles considering different layers of public health facilities to fulfil the literature gaps. Secondly, we also intended to assess whether or not public benefits are distributed relative to the needs of wealth quintiles. This study will contribute to the literature by generating comprehensive knowledge on public benefits distribution and utilization patterns on maternal and child healthcare in Bangladesh. This study will also demonstrate the efficacy with which the government allocates constrained health resources to fulfil the needs of the poor. The findings of this research will contribute to the reallocation of public resources to health services that mostly benefit the poor.

## Methods

### Data sources

This study was conducted based on the secondary data, extracted from the latest available Bangladesh Demographic and Health Survey (BDHS) 2017–18. BDHS is a cross-sectional survey that covers a nationally representative sample of households based on the multistage cluster sampling technique. A total of 20,127 ever-married women age 15–49 were interviewed from 20,250 selected households [17]. Women were asked whether they received any maternal and child healthcare services preceding the survey, and if ‘yes’, they were asked for places from where they received healthcare. Maternal health-related data were collected three years preceding the survey and child (under-five years old) health (diarrhoea, acute respiratory infection) data were collected two weeks preceding the survey respectively. In this study, we consider only selected public health facilities (primary and secondary level) for conducting BIA. To perform BIA, unit costs of services were extracted from the report of “*The Costs of the Bangladesh Essential Health Service Package: Fourth Health Population and Nutrition Sector Programme*” conducted by the Health Economics Unit (HEU), MOHFW and WHO Bangladesh. This report provides gross and unit costs for Essential Health Service Packages based on healthcare facilities or service delivery channels, with costs calculated using an ingredients-based costing approach [18].

### Socioeconomic status quintiles (wealth quintiles)

Socioeconomic status was measured by assets-based wealth index. In BDHS, data related to household assets such as availability of radio, television, mobile phone, refrigerator, almirah [a local item of furniture like a cabinet or wardrobe], water pump, and computer; household characteristics such as sources of drinking water, toilet types, cooking fuel types, and household floor, roof, and wall materials were collected from each of the households. We generated scores using Principal Component Analysis (PCA), a statistical method based on the stated durable assets and household characteristics. Households were then categorized into five equal quintiles—poorest (lower 20%), poorer, middle, richer, and richest (upper 20%) followed by generated scores.

### Benefit incidence analysis

This study focused on subsided maternal and child healthcare services in Bangladesh. In this study, we included both primary and secondary level of health facilities those are mostly provide maternal and child healthcare services in Bangladesh. Utilizing the BIA, the distribution of maternal and child healthcare benefits utilized by various wealth quintiles have been determined.

The following steps have been involved to perform BIA in our study-

1. The measures of living standards have been calculated based on asset ownership to segregate people from the lowest to the highest levels of wealth.
2. Maternal and child healthcare utilization rates across the wealth quintiles have been estimated by types and levels of services at public healthcare facilities.
3. Unit costs for healthcare services have been gathered from published literature by type of healthcare facilities.
4. Benefits were expressed in monetary values by multiplying unit costs and utilization rates for healthcare service types across the wealth quintiles.
5. The total monetary value of the benefits of overall maternal and child healthcare services was also calculated by adding all the benefits received by different wealth quintiles.
6. Finally, we compared the distribution of public benefits on maternal and child healthcare services relative to the needs of the population. In this study, need is defined as a desire for health care by individual [10].

### Concentration curve and concentration index

Concentration curves (CCs) were generated for each type of services from the selected public healthcare facilities. CC helps to explore the pattern and magnitude of inequity among wealth quintiles. The underlying mechanism of constructing CC is that CC plots cumulative rank proportion of service utilization on the vertical axis and cumulative rank proportion of population by wealth quintiles on the horizontal axis. If the concentration curve lies above the equity line, then service utilization will concentrate among the poor, meaning that poor people receive more benefits from the public facilities. On the contrary, if utilization is more concentrated among the rich people, then curve lies below the equity line. If no inequity exists, then the curve lies on the 45-degree equity line that means perfect equity among the wealth quintiles. The Concentration Index (CI) measures the gap between equity line and concentration curves. CI is twice the corresponding area between the equity line and concentration curve. The value of CI lies between − 1 and + 1. A positive CI indicates that utilization more concentrated among rich people, and a negative CI indicates utilization more concentrated among poor people. A zero (0) concentration index indicates perfect equity among wealth quintiles for receiving public benefits. We have calculated concentration indices for each of the services according to the facility types.

## Results

### Maternal and child healthcare-seeking behaviour

**Fig. 1:**
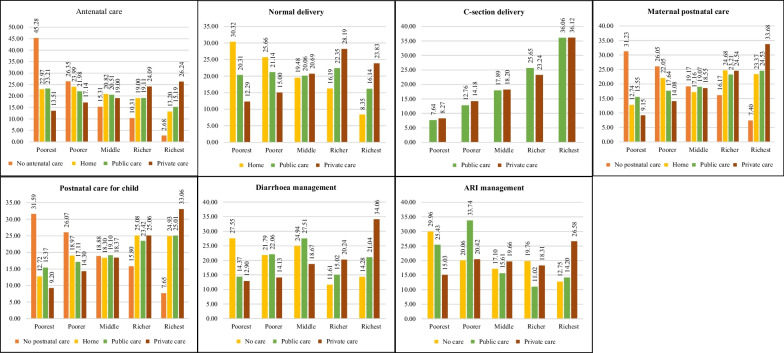
Maternal and child healthcare-seeking behaviour among wealth quintiles

Maternal and child healthcare-seeking behaviour varies across the wealth quintiles in Bangladesh (Fig. 1). Non-utilization of antenatal care (45.3% vs. 2.7%) and home care (23% vs. 13.2%) was higher among the poorest compared to the richest. A higher proportion of those in the poorest quintile received antenatal care from public facilities than the richest quintile (23.2% vs. 15.2%). On the other hand, the richest utilized more antenatal care from private sector (26.2% vs. 13.5%) than poorest. Home deliveries were also prominent among the poorest group compared to the richest (30.3% vs. 8.4%), while the poorest utilized only 12.3% from private sector for normal delivery. On the other hand, C-section deliveries from both public (36.1% vs. 7.6%) and private facilities (36.1% vs. 8.3%) were more utilized by the richest quintile than poorest. Socio economically better-off families preferred C-section deliveries rather than normal deliveries. The prevalence of not seeking maternal postnatal care (31.2% vs. 7.4%) and postnatal care for child (31.6% vs. 7.7%) was higher among the poorest quintiles compared to richest.

A similar pattern of health-seeking behaviour for diarrhoea and ARI was illustrated among wealth quintiles. Non-utilization of healthcare for diarrhoea (27.6%) and ARI (30%) was higher among poorest quintile although children from poorest households suffer more from these diseases [19]. Like the maternal care, poorest group sought child healthcare mostly from public facilities (diarrhoea, 14.4% and ARI, 25.4%), whereas rich people received more care from the private sector (diarrhoea, 34.1% and ARI, 26.6%). Finally, we may state that, poorest household considerably seek less maternal and child healthcare and they have limited access to the private health facilities than the richest.

### Utilization of maternal and child healthcare from public facilities

Table 1 shows the distribution of subsidized maternal and child healthcare benefits utilization from public health facilities across wealth quintiles in Bangladesh.

**Table 1:** Free and subsidized maternal and child healthcare benefits utilization from public health facilities, by wealth quintiles

Maternal and child healthcare services	Facility type						Overall
	Secondary level		Primary level				
	District Hospital (DH)	Maternal and Child Welfare Centre (MCWC)	Upazila Health Complex (UHC)	Upazila Health & Family Welfare Centre (UH&FWC)	Satellite Clinic and Expanded Programme on Immunization outreach (SC & EPI outreach)	Community Clinic (CC)	
**Antenatal care (Per ANC visit)**							
Wealth quintiles							
Poorest	33 (15.25)	19 (10.16)	163 (25.34)	84 (27.42)	29 (35.41)	97 (30.83)	425 (24.23)
Poorer	54 (24.55)	17 (9.10)	144 (22.46)	81 (26.43)	19 (23.58)	77 (24.45)	392 (22.35)
Middle	43 (19.68)	37 (19.37)	147 (22.85)	59 (19.12)	16 (20.27)	78 (24.84)	380 (21.66)
Richer	61 (27.76)	42 (22.13)	113 (17.51)	56 (18.16)	14 (17.40)	53 (16.87)	339 (19.33)
Richest	28 (12.76)	75 (39.24)	76 (11.84)	27 (8.86)	3 (3.33)	9 (3.01)	218 (12.43)
Concentration index	0.006	0.297	− 0.117	− 0.170	− 0.271	− 0.243	− 0.096
Total	219 (12.49)	190 (10.83)	643 (36.66)	307 (17.50)	81 (4.62)	314 (17.90)	1754
**Child-birth delivered normally**							
Wealth quintiles							
Poorest	18 (16.97)	6 (8.70)	54 (26.61)	12 (38.72)	–	1 (30.03)	91 (22.3)
Poorer	21 (19.80)	13 (19.10)	48 (24.01)	9 (29.93)	–	2 (69.97)	93 (22.79)
Middle	23 (22.10)	10 (14.42)	45 (22.50)	6 (18.90)	–	–	84 (20.59)
Richer	26 (24.81)	20 (30.12)	42 (20.81)	4 (12.46)	–	–	92 (22.55)
Richest	17 (16.32)	19 (27.66)	12 (6.06)	–	–	–	48 (11.76)
Concentration index	0.025	0.206	− 0.166	− 0.368	–	− 0.519	− 0.074
Total	105 (25.74)	68 (16.67)	201 (49.26)	31 (7.6)	-	3 (0.74)	408
**Child-birth delivered by C-section**							
Wealth quintiles							
Poorest	8 (9.74)	1 (2.41)	5 (13.25)	–	–	–	14 (8.70)
Poorer	16 (20.07)	4 (9.31)	1 (3.77)	–	–	–	21 (13.04)
Middle	15 (18.55)	5 (9.62)	11 (31.15)	–	–	–	31 (19.25)
Richer	29 (36.46)	8 (17.21)	11 (33.78)	–	–	–	48 (29.81)
Richest	12 (15.18)	29 (61.45)	6 (18.06)	–	–	–	47 (29.19)
Concentration index	0.120	0.511	0.168	–	–	–	0.156
Total	80 (49.69)	47 (29.19)	34 (21.12)	–	–	–	161
**Maternal postnatal care**							
Wealth quintiles							
Poorest	27 (14.61)	8 (6.21)	57 (23.35)	13 (34.11)	–	1 (30.03)	106 (17.88)
Poorer	38 (20.54)	17 (13.89)	51 (21.13)	13 (33.07)	–	2 (69.97)	121 (20.40)
Middle	36 (19.6)	19 (14.80)	56 (23.24)	6 (15.85)	–		117 (19.73)
Richer	51 (27.75)	31 (24.80)	54 (22.35)	5 (12.90)	–		141 (23.78)
Richest	32 (17.51)	50 (40.29)	24 (9.93)	2 (4.07)	–		108 (18.21)
Concentration index	0.063	0.325	− 0.092	− 0.309	–	− 0.519	0.027
Total	184 (31.03)	125 (21.08)	242 (40.81)	39 (6.58)	–	3 (0.51)	593
**Postnatal care for child**							
Wealth quintiles							
Poorest	25 (12.87)	9 (7.09)	63 (24.28)	13 (38.01)	–	1 (11.06)	111 (17.82)
Poorer	40 (20.47)	16 (12.70)	52 (20.02)	10 (29.69)	–	2 (25.77)	120 (19.26)
Middle	39 (19.72)	16 (12.92)	61 (23.53)	6 (18.61)	–	4 (63.17)	126 (20.22)
Richer	59 (29.72)	32 (25.30)	56 (21.64)	4 (13.01)	–	–	151 (24.24)
Richest	34 (17.22)	53 (41.99)	27 (10.54)	1 (0.68)	–	–	115 (18.46)
Concentration index	0.082	0.338	− 0.093	− 0.354	–	− 0.182	0.035
Total	197 (31.62)	125 (20.06)	260 (41.73)	34 (5.46)	–	7 (1.12)	623
**Diarrhoea management**							
Wealth quintiles							
Poorest	1 (6.3)	–	5 (17.47)	2 (34.74)	–	–	8 (15.69)
Poorer	1 (9.27)	–	7 (27.23)	1 (23.23)	–	1 (50.00)	10 (19.61)
Middle	3 (18.21)	2 (61.28)	8 (31.55)	2 (42.02)	–	1 (50.00)	16 (31.37)
Richer	3 (23.48)	–	3 (11.33)	–	–	–	6 (11.76)
Richest	6 (42.74)	1 (38.72)	4 (12.42)	–	–	–	11 (21.57)
Concentration index	0.383	0.321	− 0.056	− 0.350	–	− 0.138	− 0.238
Total	14 (27.45)	3 (5.88)	27 (52.94)	5 (9.80)	–	2 (3.92)	51
**Acute respiratory infection management**							
Wealth quintiles							
Poorest	–	–	5 (30.61)	3 (35.53)	–	1 (17.00)	9 (24.32)
Poorer	1 (25.00)	–	2 (14.03)	4 (39.28)	–	6 (83.00)	13 (35.14)
Middle	1 (25.00)	–	5 (32.13)	–	–	–	6 (16.22)
Richer	1 (25.00)	–	2 (10.06)	2 (25.19)	–	–	5 (13.51)
Richest	1 (25.00)	1 (100.00)	2 (13.17)	–	–	–	4 (10.81)
Concentration index	0.384	0.877	− 0.036	− 0.092	–	− 0.223	− 0.224
Total	4 (10.81)	1 (2.70)	16 (43.24)	9 (24.32)	–	7 (18.92)	37
**EPI vaccines (all basic vaccines)**							
Wealth quintiles							
Poorest	N/A	N/A	N/A	N/A	N/A	N/A	323 (19.97)
Poorer	N/A	N/A	N/A	N/A	N/A	N/A	336 (20.8)
Middle	N/A	N/A	N/A	N/A	N/A	N/A	308 (19.07)
Richer	N/A	N/A	N/A	N/A	N/A	N/A	323 (19.98)
Richest	N/A	N/A	N/A	N/A	N/A	N/A	326 (19.71)
Concentration index							0.005
Total							1616

Antenatal care utilization rates from the secondary level of health facilities (district hospitals (DH) and maternal and child welfare centre (MCWC)) were higher among the upper two quintiles than lower two quintiles. On the contrary, antenatal care utilization was higher among the poorest quintiles from the primary level of health facilities (UHC, UH&FWC, SC& EPI outreach, and CC). Utilization rates were 25.3% versus 11.8% from UHC, 27.4% versus 8.7% from UH&FWC, 35.4% versus 3.3% from SC& EPI outreach, and 30.8% vs. 3% from CC among the poorest vs. richest quintiles, respectively. Majority of the households from the poorest quintiles received normal delivery services from the DH, UHC, UH&FWC and CC than the richest quintiles. However, the only exception was MCWC, from where rich people received more services than poor. Household from richest quintile received more C-section delivery services from the public health facilities DH (15.2% vs. 9.7%), MCWC (61.5% vs. 2.4%) and UHC (18.1% vs. 13.3%) than the poorest. For maternal postnatal care, the richest utilized more from the DH (17.5% vs. 14.6%) and MCWC (40.3% vs. 6.2%) than the poorest, while the poorest utilized more care from the UHC (23.4% vs. 9.9%) and UH&FWC (34.1% vs. 4.1%) than the richest. A similar trend of postnatal care seeking for child was also observed among the wealth quintiles. The majority of the socioeconomically well-off families received childhood diarrhoea treatment from secondary level health facilities, while the poorest sought care mostly from primary level healthcare facilities.

### Benefit incidence analysis

**Fig. 2:**
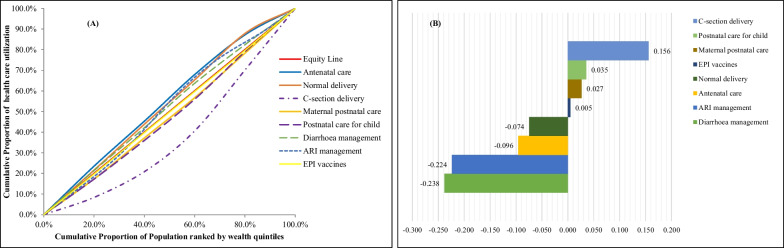
National-level public healthcare utilization (**A**) concentration curves and (**B**) concentration indices

Figure 2 illustrates (A) concentration curves and (B) concentration indices for national-level maternal and child healthcare utilization from public facilities in Bangladesh according to the types of healthcare. Concentration curves for C-section delivery, maternal postnatal care, postnatal care for child, and EPI vaccine utilization went below the equity line revealed that C-section delivery, maternal postnatal care, postnatal care for child, and EPI vaccine utilization were more concentrated among the rich people. In keeping with this finding, concentration indices for C-section delivery, maternal postnatal care, postnatal care for child, and EPI vaccine utilization were found positive which also signify that these four healthcare services were more concentrated among the rich people from public facilities. On the other hand, concentration curves for normal delivery, antenatal care, ARI, and diarrhoea treatment utilization are above the equity line and are associated with negative concentration indices. That means normal delivery, antenatal care, ARI, and diarrhoea treatment utilization from public facilities were more concentrated among the poor. Hence, we may say that maternal and child healthcare utilization from public facilities is not equitable among wealth quintiles.

**Fig. 3:**
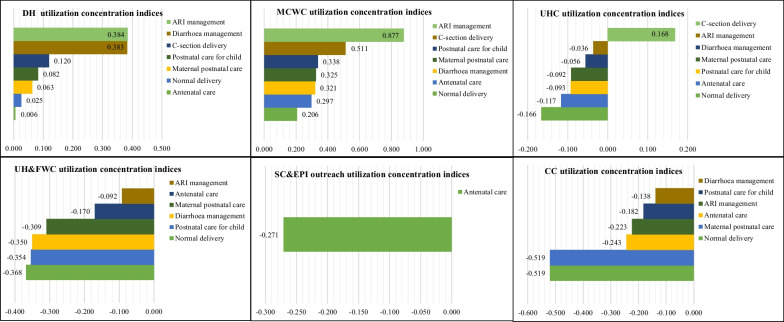
Maternal and child health utilization concentration indices, by facility types

Figure 3 depicted concentration indices for healthcare utilization from public facilities for maternal and child healthcare services according to the healthcare facilities. This figure provided an in-depth picture of inequity for maternal and child health care utilization. From the figure, it is notable that maternal and child healthcare services utilization are not equitable for either type of healthcare or level of facilities. Benefit utilization from the secondary-level facilities (DH and MCWC) are completely pro-rich that means people from the upper wealth quintiles received more benefits from DH and MCWC compared with the lower quintiles. Concentration indices for all maternal and child healthcare indicators were found positive for DH and MCWC indicated that healthcare utilization from the DH and MCWC were highly concentrated among the upper quintiles.

On the other hands, maternal and child healthcare utilization from the primary-level facilities (UHC, UH&FWC, SC&EPI outreach, and CC) is pro-poor, implying that poor household received more maternal and child healthcare services from the primary-level facilities than rich household. Concentration indices for maternal and child healthcare services for primary-level facilities were negative, except for C-section delivery from the UHC (CI = 0.168, indicates a pro-rich utilization) (Table 2).

**Table 2:** Estimated unit costs for maternal and child health services in public healthcare facilities (in BDT)

Maternal and child healthcare services	Facility type						Average cost
	Secondary level		Primary level				
	District Hospital (DH)	Maternal and Child Welfare Centre (MCWC)	Upazila Health Complex (UHC)	Upazila Health & Family Welfare Centre (UH&FWC)	Satellite Clinic and Expanded Programme on Immunization outreach (SC & EPI outreach)	Community Clinic (CC)	
Antenatal care	431.0	259.0	434.7	298.0	154.5	236.5	341.4
Child-birth delivered normally	726.6	823.1	727.9	653.2	–	631.4	677.9
Child-birth delivered by C-section	4451.0	4589.3	4451.0	–	–	–	4453.6
Maternal postnatal care	1221.0	596.3	979.6	203.4	28.8	–	644.3
Postnatal care for child	125.6	136.8	125.5	90.5	–	84.3	107.5
Diarrhoea management	259.3	95.4	255.6	87.8	–	24.6	256.4
Acute respiratory infection management	1294.2	250.8	1211.3	36.7	–	34.7	1233.0
EPI vaccines	1923.1	1907.8	1926.8	1907.8	1906.0	1906.0	1873.8

*Source*: The costs of the Bangladesh Essential Health Service Package: Fourth Health Population and Nutrition Sector Programme, 2018

The monetary amount (in BDT) of total benefits received for each of the maternal and child healthcare services from public facilities according to wealth quintiles is presented in Table 3. Total benefits in monetary units were calculated based on the unit costs for maternal and child health services (Table 2) and utilization rates.

**Table 3:** Distribution of total benefits received from public facilities, by wealth quintiles (in BDT)

Maternal and child healthcare services	Facility type						Overall
	Secondary level		Primary level				
	District Hospital (DH)	Maternal and Child Welfare Centre (MCWC)	Upazila Health Complex (UHC)	Upazila Health & Family Welfare Centre (UH&FWC)	Satellite Clinic and Expanded Programme on Immunization outreach (SC & EPI outreach)	Community Clinic (CC)	
**Antenatal care (Per ANC visit)**							
Wealth quintiles							
Poorest	431*33 = 14,221	259*19 = 4921	434.7*163 = 70,851	298*84 = 25,032	154.5*29 = 4479	236.5*97 = 22,936	341.4*425 = 145,082
Poorer	431*54 = 23,271	259*17 = 4403	434.7*144 = 62,592	298*81 = 24,138	154.5*19 = 2935	236.5*77 = 18,207	341.4*392 = 133,817
Middle	431*43 = 18,531	259*37 = 9584	434.7*147 = 63,896	298*59 = 17,582	154.5*16 = 2471	236.5*78 = 18,443	341.4*380 = 129,721
Richer	431*61 = 26,288	259*42 = 10,879	434.7*113 = 49,118	298*56 = 16,688	154.5*14 = 2162	236.5*53 = 12,532	341.4*339 = 115,724
Richest	431*28 = 12,067	259*75 = 19,427	434.7*76 = 33,035	298*27 = 8046	154.5*3 = 463	236.5*9 = 2128	341.4*218 = 74,419
**Child-birth delivered normally**							
Wealth quintiles							
Poorest	726.6*18 = 13,079	823.1*6 = 4939	727.9*54 = 39,307	653.2*12 = 7838	–	631.4*1 = 631	677.9*91 = 61,689
Poorer	726.6*21 = 15,259	823.1*13 = 10,700	727.9*48 = 34,939	653.2*9 = 5879	–	631.4*2 = 1263	677.9*93 = 63,045
Middle	726.6*23 = 16,712	823.1*10 = 8231	727.9*45 = 32,756	653.2*6 = 3919	–	–	677.9*84 = 56,944
Richer	726.6*26 = 18,892	823.1*20 = 16,462	727.9*42 = 30,572	653.2*4 = 2613	–	–	677.9*92 = 62,367
Richest	726.6*17 = 12,352	823.1*19 = 15,639	727.9*12 = 8735	–	–	–	677.9*48 = 32,539
**Child-birth delivered by C-section**							
Wealth quintiles							
Poorest	4451*8 = 35,608	4589.3*1 = 4589	4451*5 = 22,255	–	–	–	4453.6*14 = 62,350
Poorer	4451*16 = 71,216	4589.3*4 = 18,357	4451*1 = 4451	–	–	–	4453.6*21 = 93,526
Middle	4451*15 = 66,765	4589.3*5 = 22,947	4451*11 = 48,961	–	–	–	4453.6*31 = 138,062
Richer	4451*29 = 129,079	4589.3*8 = 36,714	4451*11 = 48,961	–	–	–	4453.6*48 = 213,773
Richest	4451*12 = 53,412	4589.3*29 = 133,090	4451*6 = 26,706	–	–	–	4453.6*47 = 209,319
**Maternal postnatal care**							
Wealth quintiles							
Poorest	1221*27 = 32,967	596.3*8 = 4770	979.6*57 = 55,837	203.4*13 = 2644	–	28.8*1 = 29	644.3*106 = 68,296
Poorer	1221*38 = 46,398	596.3*17 = 10,137	979.6*51 = 49,960	203.4*13 = 2644	–	28.8*2 = 54	644.3*121 = 77,960
Middle	1221*36 = 43,956	596.3*19 = 11,330	979.6*56 = 54,858	203.4*6 = 1220	–	–	644.3*117 = 75,383
Richer	1221*51 = 62,271	596.3*31 = 18,485	979.6*54 = 52,898	203.4*5 = 1017	–	–	644.3*141 = 90,846
Richest	1221*32 = 39,072	596.3*50 = 29,815	979.6*24 = 23,510	203.4*2 = 407	–	–	644.3*108 = 69,584
**Postnatal care for child**							
Wealth quintiles							
Poorest	125.6*25 = 3140	136.8*9 = 1231	125.5*63 = 7907	90.5*13 = 1177	–	84.3*1 = 84	107.5*111 = 11,933
Poorer	125.6*40 = 5024	136.8*16 = 2189	125.5*52 = 6526	90.5*10 = 905	–	84.3*2 = 169	107.5*120 = 12,900
Middle	125.6*39 = 4898	136.8*16 = 2189	125.5*61 = 7656	90.5*6 = 543	–	84.3*4 = 337	107.5*126 = 13,545
Richer	125.6*59 = 7410	136.8*32 = 4378	125.5*56 = 7028	90.5*4 = 362	–	–	107.5*151 = 16,233
Richest	125.6*34 = 4270	136.8*53 = 7250	125.5*27 = 3389	90.5*1 = 91	–	–	107.5*115 = 12,363
**Diarrhoea management**							
Wealth quintiles							
Poorest	259.3*1 = 259	–	255.6*5 = 1278	87.8*2 = 176	–	–	256.4*8 = 2051
Poorer	259.3*1 = 259	–	255.6*7 = 1789	87.8*1 = 88	–	24.6*1 = 25	256.4*10 = 2564
Middle	259.3*3 = 778	95.4*2 = 191	255.6*8 = 2045	87.8*2 = 176	–	24.6*1 = 25	256.4*16 = 4102
Richer	259.3*3 = 778	–	255.6*3 = 767	–	–	–	256.4*6 = 1538
Richest	259.3*6 = 1,556	95.4*1 = 95	255.6*4 = 1022	–	–	–	256.4*11 = 2820
**Acute Respiratory Infection management**							
Wealth quintiles							
Poorest	–	–	1211.3*5 = 6057	36.7*3 = 110	–	34.8*1 = 35	1233*9 = 11,097
Poorer	1294.2*1 = 1294	–	1211.3*2 = 2423	36.7*4 = 147	–	34.8*1 = 209	1233*13 = 16,029
Middle	1294.2*1 = 1294	–	1211.3*5 = 6057	–	–	–	1233*6 = 7398
Richer	1294.2*1 = 1294	–	1211.3*2 = 2423	36.7*2 = 73	–	–	1233*5 = 6165
Richest	1294.2*1 = 1294	250.8*1 = 251	1211.3*2 = 2423	–	–	–	1233*4 = 4932
**EPI vaccines (all basic vaccines)**							
Wealth quintiles							
Poorest	N/A	N/A	N/A	N/A	N/A	N/A	1873.8*323 = 605,237
Poorer	N/A	N/A	N/A	N/A	N/A	N/A	1873.8*336 = 629,597
Middle	N/A	N/A	N/A	N/A	N/A	N/A	1873.8*308 = 577,130
Richer	N/A	N/A	N/A	N/A	N/A	N/A	1873.8*323 = 605,237
Richest	N/A	N/A	N/A	N/A	N/A	N/A	1873.8*326 = 610,859

Each cell calculated by multiplying unit costs with number of healthcare utilization

National-level benefits for maternal and child healthcare services are calculated by adding all monetary benefits from the each of the healthcare services across the wealth quintiles (Table 4). Higher proportion of national-level monetary benefits from DH and MCWC was received by the upper two quintiles (richest 15.8% and richer 31.3% from DH) and (richest 49.8% and richer 21% from MCWCs), compared to lower two quintiles (poorest 12.7% and poorer 20.7% from DH) and (poorest 5% and poorer 11.1% from MCWC). On the contrary, higher monetary benefits was received by the households from poorest quintiles from the UHC, UH&FWC, SC&EPI outreach, and CC. The proportion of benefits received was 23.3% versus 11.3%, 29.9% versus 6.9%, 35.8% versus 3.7% and 30.8% versus 2.8% among poorest vs. richest quintiles from the UHC, UH&FWC, SC&EPI outreach, and CC respectively. However, the overall monetary benefits for maternal and child healthcare services from public facilities was received mostly by the socioeconomically wealthier (upper two quintiles) household. The proportion of benefits received by upper two quintiles (richest 19.8% and richer 21.7%) is more than that of lower two quintiles (poorest 18.9% and 20.1%).

**Table 4:** Distribution of national-level total maternal and child healthcare benefits received from public facilities, by wealth quintiles (in BDT)

Wealth quintiles	Facility type, total (%)						Overall, total (%)
	Secondary level		Primary level				
	District Hospital (DH)	Maternal and Child Welfare Centre (MCWC)	Upazila Health Complex (UHC)	Upazila Health & Family Welfare Centre (UH&FWC)	Satellite Clinic and Expanded Programme on Immunization outreach (SC & EPI outreach)	Community Clinic (CC)	
Poorest	99,274 (12.65)	20,451 (4.95)	203,491 (23.31)	36,977 (29.94)	4479 (35.80)	23,715 (30.75)	967,735 (18.87)
Poorer	162,722 (20.73)	45,787 (11.08)	162,680 (18.63)	33,801 (27.37)	2935 (23.46)	19,931 (25.85)	1029,438 (20.07)
Middle	152,934 (19.48)	54,471 (13.18)	216,227 (24.77)	23,440 (18.98)	2471 (19.75)	18,805 (24.39)	1002,285 (19.54)
Richer	246,012 (31.34)	86,918 (21.04)	191,766 (21.97)	20,753 (16.80)	2162 (17.28)	12,532 (16.25)	1111,883 (21.68)
Richest	124,023 (15.80)	205,567 (49.75)	98,820 (11.32)	8544 (6.92)	463 (3.70)	2128 (2.76)	1016,835 (19.83)

### Assessment of distribution of benefits

**Fig. 4:**
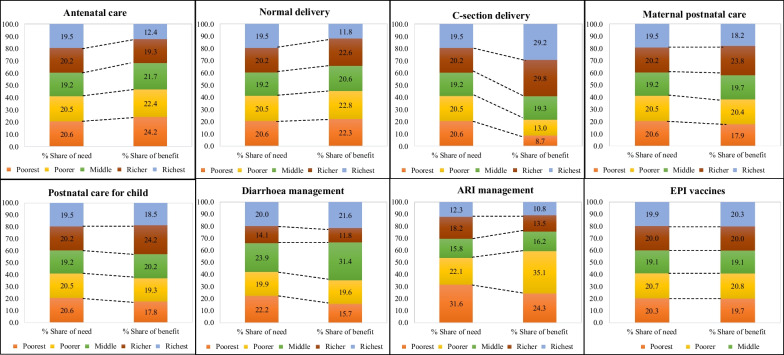
Distribution of total benefits in comparison with need for healthcare

The percentage share of needs and benefits across the different wealth quintiles by public healthcare services is presented in Fig. 4. Unequal utilization of public benefits among wealth quintiles observed in Bangladesh while distributions of benefits were not even according to their needs. Households from lower quintiles unable to manage private healthcare due to high OOP payments, but they suffer more from all kind of health hazards because of poor hygiene, less education, lack of awareness, poor housing conditions, etc., therefore, public benefits should be allocated concentrating them [20]. Our focus in this part is to assess the distribution of public benefits from the point of lower quintiles because in Bangladesh private care utilization is higher among upper quintiles as they have enough money to seek healthcare privately (Fig. 1).

In Bangladesh, although poorest quintile has more health care needs for maternal and child healthcare services, share of benefits was less than their share of needs in most cases except antenatal care and normal delivery. Surprisingly, poorest, i.e. lowest 20% household, cannot access 20% share of public benefits in most of the maternal and child healthcare services even if we ignore their needs. The percentage shares of needs and benefits among poorest quintiles were (20.6% vs. 8.7% for C-section delivery), (20.6% vs. 17.9% for maternal postnatal care), (20.6% vs. 17.8% for postnatal care for child), (22.2% vs 15.7% for diarrhoea), (31.6% vs. 24.3% for ARI) and (20.3% vs. 19.7% for EPI vaccines). In contrast, for antenatal care and normal delivery, the households from poorest quintile utilized more public benefits than their needs (20.6% needs vs. 24.2% benefits for antenatal care), and (20.6% needs vs. 22.3% benefits for normal delivery), respectively.

## Discussion

This study intended to assess equity in the distribution of public healthcare benefits across wealth quintiles, particularly in maternal and child healthcare services according to the level of public healthcare facilities in Bangladesh. The study also evaluated whether health benefits are allocated among wealth quintiles relative to their needs of healthcare or not. This study reveals several findings from the BIA based on nationally representative survey data. Study found an unequal distribution of public healthcare utilization and benefits among the wealth quintiles for maternal and child healthcare services across the different levels of public healthcare facilities. In addition, we also observed inefficient distribution of public subsidies in health, i.e. resources in the health system were not allocated relative to the needs of the population among wealth quintiles. This type of evidence is vital for efficient allocation of resources in health to achieve universal health coverage in the context of Bangladesh.

This study found that, in general, public benefits from maternal and child healthcare services in Bangladesh are pro-rich, implying that benefits from public facilities were more utilized by the upper quintiles. Previous research has also demonstrated a similar finding that rich utilize more subsidies from public healthcare facilities in Bangladesh [15]. Hence, we may say that allocation of maternal and child healthcare subsidies in Bangladesh are not particularly targeted to the poorest: rather it has consistently been in favour of wealthier. A BIA study by Bowser et al. showed that public healthcare utilization was slightly pro-rich in India [21]. Another study in South Africa also found that lower-income groups do not benefit much from public health services than higher-income groups [22]. Similarly, other BIAs in different countries illustrated the utilization of public subsidies as pro-rich [8, 9, 12, 13, 23–25]. Moreover, a systematic review of BIA from the 24 developing countries also showed that healthcare benefits in sub-Saharan Africa and Asia–Pacific is pro-rich and highly pro-rich for hospital services [26]. In contrast to the findings of our study in other LMICs, Halasa et al. [27] found that the poorest groups of the Jordanian population were the main users of public healthcare services meaning a pro-poor distribution of health benefits. Similarly, studies in Nigeria and Cambodia also demonstrated that priority public health services were well-targeted to the poorer groups and rural residence [28, 29]. The pro-rich distribution of public benefits in health could be because of lower education, lack of health knowledge, and health-seeking from informal providers among the lower quintiles in Bangladesh [20, 30]. OOPE on health including informal payments and long waiting time in public facilities could be the burdens for poor communities and possibly responsible for not receiving benefits from public facilities.

This study also shows that benefits from both DHs and MCWCs were pro-rich while benefits from UHC, UH&FWC, SC&EPI outreach, and CC tend to favour the underprivileged groups. This finding indicates that secondary levels of care are mostly concentrated among rich people, while at primary levels poor people get more access to healthcare. This result is consistent with a report by Pearson in 2002 which found that the richest mostly utilized more healthcare from the secondary and tertiary level health facilities than poorest, while poorest utilized more healthcare from primary level health facilities in Bangladesh [31]. Recent studies conducted in India and African countries have also illustrated a pro-rich distribution of benefits for higher-level public health facilities but a pro-poor distribution for lower-level public health facilities [21, 25, 28, 32, 33]. Secondary levels of health facilities (DH and MCWC) are usually located in city areas, and services are provided by the specialized healthcare providers, whereas primary levels of health facilities are in rural areas at the community level where most of the poor people live in. The pro-rich distribution at secondary levels of facilities can be explained by the financial barriers for the poor because of the high indirect OOPE on healthcare such as transportation costs, food and accommodation costs for the caregivers, and direct costs such as user fees and medicine costs that are not available at the health facility. However, another reason for the pro-rich distribution at the secondary level facility could be that urban women are more likely to utilize maternal and child healthcare than rural women in Bangladesh [19]. On the contrary, geographical access, short distance from home to the health facility, small amount or no user fees, and lower barriers attributable to indirect health expenditure could be key determinants for pro-poor distribution at primary level health facilities.

Another key conclusion of this study is that the poor have the greatest need for maternal and child healthcare services yet receive the smallest proportion of benefits: even the lowest 20% of households are unable to access 20% share of public benefits. Such findings are consistent with previous studies in India, South Africa, Uganda, and Zambia [13, 21, 22, 25, 32, 33]. Our finding is also consistent with the previous study in Bangladesh that the poorest quintiles cannot fulfil their share of needs from public subsidies [15]. That study also found that share of benefits from all type of providers including private and NGO is highly pro-rich compared to public providers. Due to the high OOPE in the private sector, the rich are more likely to seek healthcare from there because of their higher ability to pay than poor. Therefore, in the view of equity and universal health coverage, it is expected that poorer groups should get more benefits from the subsidized public healthcare services to meet their health needs.

Findings of this BIA study in Bangladesh follows the widely applicable “inverse care law” proposed by Julian Tudor Hart in 1971, which stated that people with more need for healthcare services benefit less than those who need comparatively less healthcare services [34]. The pro-rich distribution of maternal and child healthcare from public health facilities in Bangladesh should draw further attention to the policymakers. It indicates that urgent health financing reforms are required in Bangladesh to provide universal health coverage for the population, especially for the poor. The pro-poor distribution of primary and community-level health facilities illustrates that the GOB has successfully targeted primary and community-level health facilities to reach the poor people, but the overall distribution of health benefits is still pro-rich. Poor accessibility at the secondary level of health facilities implies that user fees of those facilities and indirect transportation costs reduce their access to healthcare compared to the socioeconomically well off. Therefore, burden of OOPE on health could be the main reason for unequal utilization of health benefits in Bangladesh. However, a greater share of government health spending in Bangladesh is focused on the secondary- and tertiary-level facility which is not currently accessible for the poor. This study recommends redistribution of health resources where healthcare services are mostly utilized by the poor. To ensure equitable distribution for health benefits, government should give more emphasis on lower-level health facilities. At the same time, reform is needed for the secondary- and tertiary-level health facilities to guarantee the availability and accessibility to meet health need for the poor. In addition, rapid implementation of pro-poor policies developed under the "Bangladesh Health Financing Strategy" is essential for fair health access and distribution of benefits [35]. Policymakers should concentrate on poverty reduction strategies, relaxation of user fees and social protection scheme such as social health insurance and community-based health insurance, especially among the poor vulnerable community to increase their access to health.

We used nationally representative survey data; however, this study has several limitations. First, we conducted BIA based on the maternal and child healthcare services and are not able to consider all type of healthcare services available at public facilities. Second, our study is limited to public primary and secondary level of healthcare facility, but private and nongovernment organizations (NGOs) also play a significant role in Bangladesh. Third, this study is based on government implementation costs for maternal and child healthcare services but OOPE on health at public facilities could be another interesting dimension for conducting BIA in Bangladesh. Finally, another limitation of this research is that BDHS data may be affected by recall bias, which may have an impact on the absolute numbers given in this study.

## Conclusion

Equitable health access among wealth quintiles and distribution of benefits relative to the needs of the population are the preconditions for achieving universal health coverage. Public health spending should be more concentrated on the poor and vulnerable population to increase their accessibility on health. This BIA study focused on the assessment of the equitable distribution of public subsidies on maternal and child healthcare services among wealth quintiles in Bangladesh. Our study illustrated that public health spending in Bangladesh on maternal and child healthcare services was not equally distributed among wealth quintiles. Overall monetary benefits were more utilized by the rich relative to the poor. Hence, policymakers should focus on redistribution of resources on health by targeting the socioeconomically vulnerable segments of the population, and strategies are needed to reduce reliance on OOPE and to increase accessibility of health services among the lower wealth quintiles to meet their health needs.

## Data Availability

The BDHS 2017–18 dataset is publicly available for download from the DHS Program website (https://dhsprogram.com/data/available-datasets.cfm).
